# Human ABCE1 exhibits temperature‐dependent heterologous co‐functionality in *S. cerevisiae*

**DOI:** 10.1002/2211-5463.13463

**Published:** 2022-07-19

**Authors:** Miki Wada, Koichi Ito

**Affiliations:** ^1^ Laboratory of Molecular Genetics, Department of Computational Biology and Medical Sciences, Graduate School of Frontier Sciences The University of Tokyo Chiba Japan

**Keywords:** ABCE1, heterologous functionality, ribosome recycling, *Saccharomyces cerevisiae*, temperature‐dependency, translation, RLI1

## Abstract

ABCE1 protein (Rli1 in *Saccharomyces cerevisiae*) is a unique ribosome recycling factor that is composed of an N‐terminal FeS cluster domain and two ATPase domains. Here, we report that heterologous expression of human ABCE1 in *S. cerevisiae* is unable to complement conditional knockout of ABCE1 (Rli1), at a typical experimental temperature of 30 °C. However, low but significant growth was observed at high temperature, 37 °C. Considering the close interaction of ABCE1 with translation factors and ribosomal components, the observed temperature‐dependent complementation may be attributed to heterologous co‐functionality of ABCE1 with *S. cerevisiae* factor(s), and might reflect functional upregulation of human ABCE1 at its functional temperature.

## Graphical abstract

Heterologous expression of the human ortholog of ribosome recycling factor ABCE1 in *Saccharomyces cerevisiae* failed to complement conditional knockout of ABCE1 (Rli1) at 30 °C, but significant growth recovery was observed at high temperature (37 °C). Thermo‐dependent behavior of ABCE1 variants may aid in the elucidation of details of the ribosomal recycling pathway.

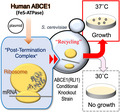

**Abbreviations**
dox: doxycycline
tet: tetracycline
WT: wild‐type

Temperature sensitivity is one of the well‐known functional assay tools in some organisms, including *Saccharomyces cerevisiae*. The temperature sensitive mutations of essential genes, which lack functionality at higher temperature, are often used for complementation assays and functional tests of heterologous gene products. In *S. cerevisiae*, temperature sensitive mutants usually show their defects at around 37 °C, contrary to typical experimental conditions at 30 °C.

ABCE1 protein (Rli1 in *S. cerevisiae*) is a unique ribosome recycling factor that is composed of N‐terminal FeS cluster domain and the following two ATPase domains [1, 2, 3]. Studies have revealed that ABCE1 binds to the ribosome after translation termination factor eRF1, and induces ribosome splitting to 40S and 60S subunits via ATPase activity [4, 5]. ABCE1 was first isolated as RNase L inhibitor in a human cell line [6]; however, this function is not considered to be broadly shared. Later, ABCE1 was found to commonly function in ribosome recycling in eukaryotes and archaea. Thus, in the present study, we use the protein name ‘ABCE1’ for both human ABCE1 and *S. cerevisiae* Rli1.

Here, we report that heterologous functionality of human ABCE1 depends on temperature in *S. cerevisiae*. It could not complement conditional knockout of *S. cerevisiae* ABCE1 at 30 °C, but showed functionality at 37 °C. This finding may provide a new functional basis with respect to heterologous functionality.

## Materials and methods

### Yeast strains and media

The *S. cerevisiae* strains used are:

BY4727: *MATα his3∆200 leu2∆0 lys2∆0 met15∆0 trp1∆63 ura3∆0*,
Y381 (tetOFF rli1) (made in the present study); *MATα his3∆200 leu2∆0 lys2∆0 met15∆0 trp1∆63 ura3∆0* tetO‐Rli1 (made in the present study), and
S19‐H06 (tetaptOFF rli1): *MATα, his3Δ200 leu2Δ0 lys2Δ0 met15Δ0 trp1Δ63 ura3Δ0 + TcApt‐TetOFF‐ TDH3p‐tc3‐3xHA‐YDR091C (RLI1)*, (made in the present study).

Synthetic complete medium was used for yeast plates, prepared with leucine dropout mix (Formedium, Swaffham, UK), supplemented with 10 μg·mL^−1^ doxycycline (dox) or 50 μg·mL^−1^ tetracycline (tet) when necessary.

### Yeast plasmids construction

The yeast plasmids used are listed in Table 1.

**Table 1:** Plasmid list.

Name	Source or construct
p415ADH	Mumberg *et al* [7].
p415GPD	Mumberg *et al* [7].
scABCE1L	p415CYC1‐PstI‐scABCE1‐SalI
scABCE1M	p415ADH‐PstI‐scABCE1‐SalI
scABCE1H	p415GPD‐PstI‐scABCE1‐SalI
HA‐scABCE1M	p415ADH‐SpeI‐HAtag‐scABCE1‐SalI
HA‐scABCE1H	p415GPD‐SpeI‐HAtag‐scABCE1‐SalI
hsABCE1M	P415ADH‐BamHI‐hsABCE1‐HindIII
hsABCE1H	P415GPD‐BamHI‐hsABCE1‐HindIII
HA‐hsABCE1M	P415ADH‐SpeI‐HAtag‐hsABCE1‐HindIII
HA‐hsABCE1H	P415GPD‐SpeI‐HAtag‐hsABCE1‐HindIII
scabce1‐L252S/S318S	P415GPD‐PstI‐scabce1 T755C/T953C‐SalI
scabce1‐L252S	P415GPD‐PstI‐scabce1 T755C‐SalI
scabce1‐L318S	P415GPD‐PstI‐scabce1 T953C‐SalI
scabce1‐S250P	P415GPD‐PstI‐scabce1 T750C‐SalI

L indicates low expression promoter (P_CYC1_); M indicates medium expression promoter (P_ADH_); H indicates high expression promoter (P_GPD_).

Human ABCE1 was PCR amplified from the Hela cDNA library (Clonetec, Mountain View, CA, USA) and cloned into the vectors with *Bam*HI and *Hind*III sites. *S. cerevisiae* ABCE1 was cloned from genomic DNA of BY4727 strain and cloned into the vectors with *Pst*I and *Sal*I sites. HA‐tagged hsABCE1 and scABCE1 were PCR amplified with HA‐tag encoding primers hsHA and scHA, and cyc1 terminator primer from each of plasmids, respectively, and cloned into the vector with indicated restriction enzyme sites (Table 1).

hsHA:CCCACTAGTATGTATCCGTATGATGTTCCGGATTATGCAGGTGGTATGAGTGATAAAAACAGTCGTATC.
scHA:CCCACTAGTATGTATCCGTATGATGTTCCGGATTATGCAGGTGGTATGGCAGACAAGTTAACGAGAATT.

### Ribosome fraction preparation

*S. cerevisiae* transformant cells were harvested, suspended with P buffer (25 mm Tris–HCl pH7.2, 50 mm KCl, 5 mm MgCl_2_ and 5 mm 2‐melcaptoethanol) and lysed with glass beads using FastPrep‐24 instrument (MP‐Biomedicals, Santa Ana, CA, USA). The lysates were separated by ultracentrifugation (CS120FNX; Hitachi, Tokyo, Japan) with a S55A2 rotor at 201,000*g* for 50 min, through a 15% sucrose cushion (15% sucrose, 25 mm Tris–HCl, 0.1 m KCl, 10 mm MgCl_2_, 2 mm dithiothreitol) to obtain the ribosome fraction as a pellet. The pellets were solved by P buffer and treated with 2 × SDS‐PAGE sample buffer. Total lysates were precipitated with 10% trichloroacetic acid solution and solved with SDS‐PAGE sample buffer.

### SDS‐PAGE and Western blot analysis

Samples were separated by SDS‐PAGE with 10% polyacrylamide gel and stained with Bio‐Safe Coomassie stain (Bio‐Rad, Hercules, CA, USA). For Western blot analysis, samples were separated by 8% polyacrylamide gel and transferred to Hybond‐ECL membrane (GE Healthcare, Chicago, IL, USA). The membrane was treated with anti‐HA polyclonal antibody (Proteintech, Rosemont, IL, USA) and anti‐rabbit IgG HRP‐linked antibody (GE Healthcare), or PGK1 monoclonal antibody (anti‐PGK 22C4D8; Thermo Fisher, Waltham, MA, USA) and anti‐mouse IgG HRP‐linked antibody (GE Healthcare). Signals were developed with ImmunoStar LD (Wako, Osako, Japan) and detected by LAS‐3000 Mini digital imaging system (Fujifilm, Tokyo, Japan).

### Isolation of *S. cerevisiae* ABCE1 mutations

The plasmid, scABCE1 gene in p415GPD was randomly mutagenized by error‐prone PCR with vector part primers. The mutagenized scABCE1 fragment and linearized p415GPD vector were introduced into Y381 strain for recombination. Transformants grown on SC‐L(leucine) plates were patched on either SC‐L or SC‐L dox at 30 and 37 °C. Colonies that show reduced growth at 37 °C were selected and analyzed.

## Results

### Confirmation of the functionality of HA‐tagged ABCE1

**Fig. 1:**
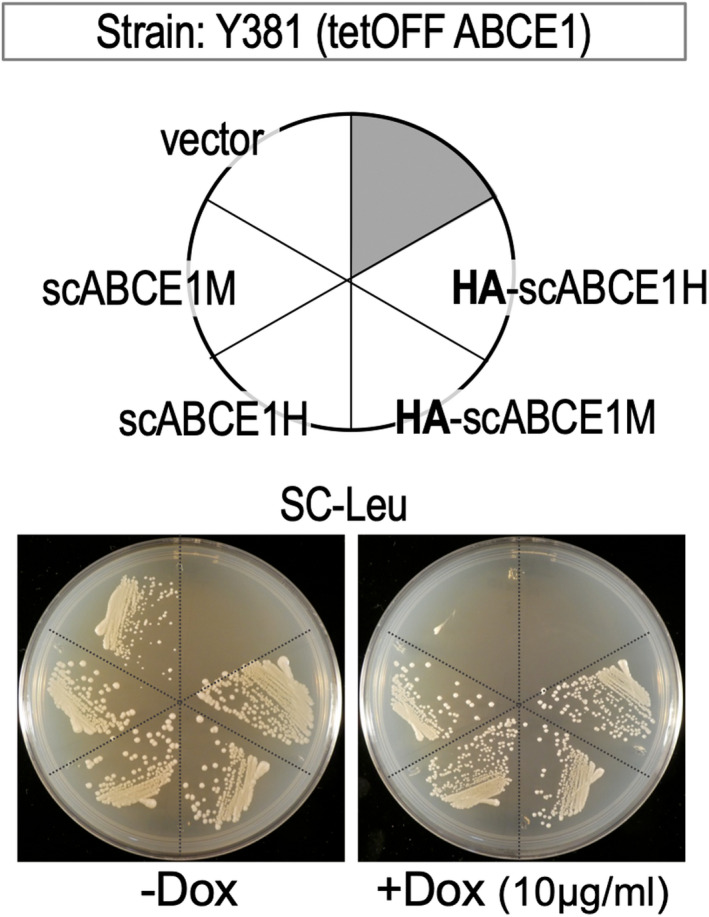
Functionality of N‐terminally HA‐tagged ABCE1. Plasmids were introduced into Y381 tetOFF ABCE1 strain and examined for growth complementation in SC‐L(leucine) plates (control, left) and SC‐L plates containing 10 μg·mL^−1^ dox (right) as being indicative of functionality. Vector: p415ADH, scABCE1M (medium expression), scABCE1H (high expression), HA‐scABCE1M (medium expression) and HA‐scABCE1H (high expression). Experiments were performed in duplicate.

N‐terminally HA‐tagged ABCE1 was prepared and its functionality was examined in *S. cerevisiae*. HA‐tag encoding sequence was fused to the scABCE1 gene, and cloned into p415ADH and p415GPD vectors to express HA‐tagged scABCE1 (HA‐scABCE1M and HA‐scABCE1H; medium and high expression promoters, respectively). The functionality of HA‐scABCE1 was examined by introducing the plasmids into a conditional knockout strain, Y381 tetOFF ABCE1(Rli1). In the presence of 10 μg·mL^−1^ dox, HA‐scABCE1 supported transformant growth comparable to scABCE1 expressed from the same vectors (Fig. 1).

### Expression and ribosomal binding of human ABCE1 (hsABCE1)

**Fig. 2:**
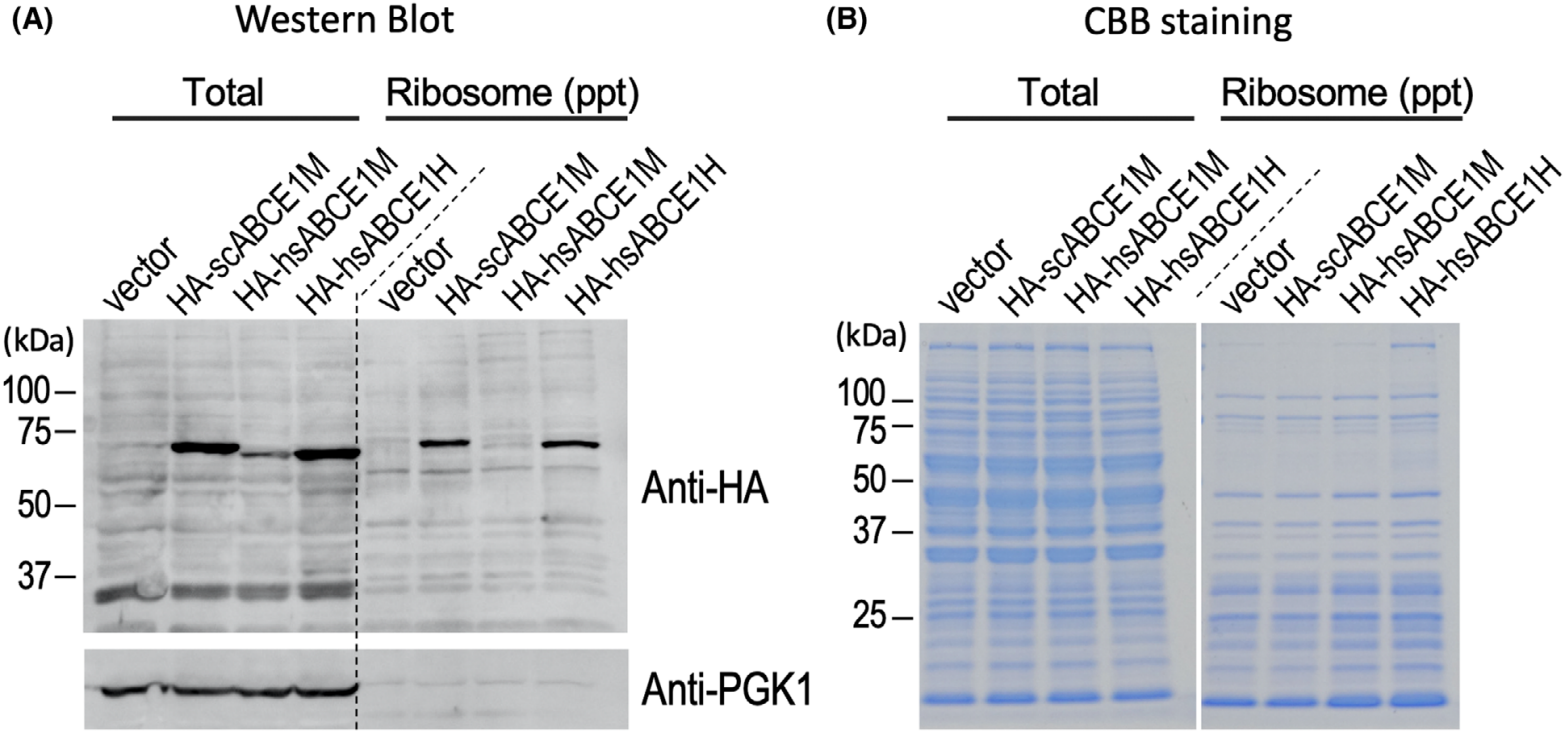
Expression and ribosome binding of scABCE1 and human ABCE1 (hsABCE1). (A) Lysates from the plasmid transformants of WT strain were separated by 8% SDS‐PAGE gel and examined with anti‐HA antibody by Western blot analysis (left, total). Vector: p415ADH, HA‐scABCE1M, HA‐hsABCE1M and HA‐hsABCE1H. The ribosome fractions were prepared from the lysates through a 15% sucrose cushion and the existence of HA‐tagged ABCE1s in the ribosome was examined with anti‐HA antibody by Western blot analysis [right, ribosome (ppt)]. The applied sample equivalent ratio of total : ribosome was 1 : 5. The same filters were detected with anti‐PGK1 as a control. Experiments were performed in duplicate to confirm reproducibility. (B) Coomassie brilliant blue staining of the same samples in (A) separated by 10% SDS‐PAGE gel.

In wild‐type (WT) BY4727 strain, expression of HA‐scABCE1 and HA‐hsABCE1 (HA‐tagged human ABCE1) was examined by Western blot analysis with anti‐HA antibody. The results confirmed expression of HA‐scABCE1 and HA‐hsABCE1 (Fig. 2A, upper left). However, hsABCE1 expression was far lower than scABCE1 from the same vector promoter. Instead, the expression level of hsABCE1 from p415GPD (hsABCE1H) is similar to that of scABCE1 from p415ADH vector (scABCE1M). To confirm the presence of ABCE1 in the ribosome, and to the examine binding of hsABCE1 to the ribosome, ribosome fractions were obtained by ultracentrifugation through a 15% sucrose cushion and analyzed by Western blotting. Both scABCE1 and hsABCE1 were found in the ribosome fraction, reflecting the expression level in total lysate (Fig. 2A, upper right). The filters were detected with anti‐PGK1 antibody as a cytosolic control marker (Fig. 2A, lower). The proteins of total lysates and ribosome fractions were visualized by Coomassie brilliant blue staining (Fig. 2B). The ribosomal fractions showed small ribosomal protein size bands collected through the sucrose cushion as ribosomal complexes pellets.

### Temperature‐dependent co‐functionality

**Fig. 3:**
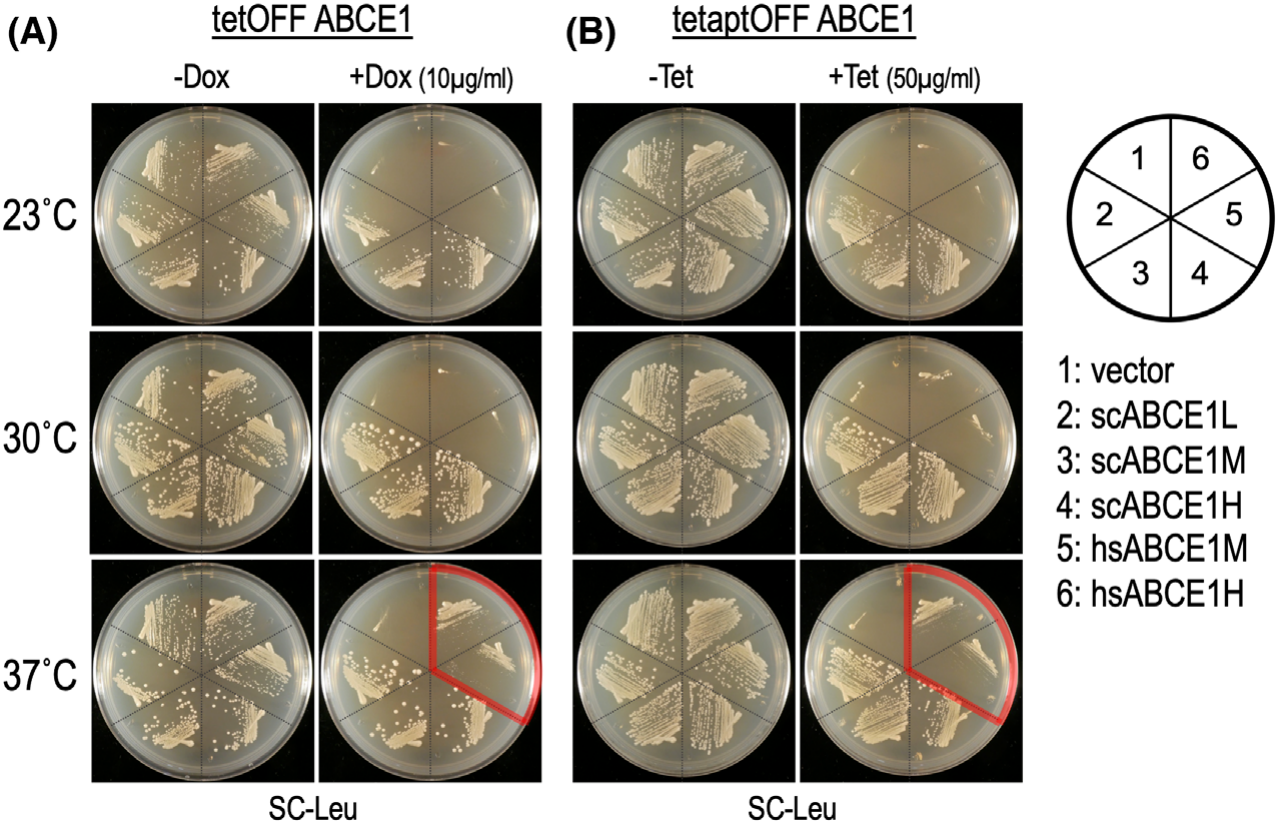
Complementation analysis of conditional ABCE1 knockout strains by hsABCE1. (A) The plasmids, p415ADH, scABCE1L, scABCE1M, scABCE1H, hsABCE1M and hsABCE1H, were introduced into Y381 tetOFF ABCE1 strain. The transformants were examined for growth on control SC‐L plates (control, left) and SC‐L plates containing 10 μg·mL^−1^ dox (right) as being indicative of functionality at 23, 30 and 37 °C. (B) Plasmids were introduced into S19‐H06 tetaptOFF strain and the transformants were examined for growth on SC‐L plates (control, left) and SC‐L plates containing 50 μg·mL^−1^ tetracycline (right) as being indicative of functionality at 23, 30 and 37 °C. Each experiment was performed in duplicate and repeated three times.

Functional exchangeability of hsABCE1 with scABCE1 was examined in two conditional knockout strains of ABCE1: Y381, as described above, and S19‐H06 tetaptOFF strain, in which ABCE1 expression from tetracycline aptamer‐containing promoter is regulated by tetracycline with respect to translation level [8]. The results show that, at 30 °C, hsABCE1 was unable to complement either of the strains Y381(Fig. 3A) and S19‐H06 (Fig. 3B). Growth at 23 °C appears to be generally slower growth than at 30 °C, reflecting the effect of temperature. Intriguingly, at 37 °C, hsABCE1 showed slight but significant complementation of lack of scABCE1 (Fig. 3), dependent on expression level.

This unusual temperature‐dependent functionality of heterologous hsABCE1 is assumed to be based on its co‐functionality in the translation process on the ribosome of *S. cerevisiae*. ABCE1 undergoes complex interaction changes in the ribosome with and without its ATPase activities. Considering that the ribosomes are essential machinery for life, their optimal functional temperature would be affected by the physiology of an organism. Thus, the functionality of hsABCE1 in *S. cerevisiae* might reflect their better co‐function at 37 °C.

### scABCE1 mutants

**Fig. 4:**
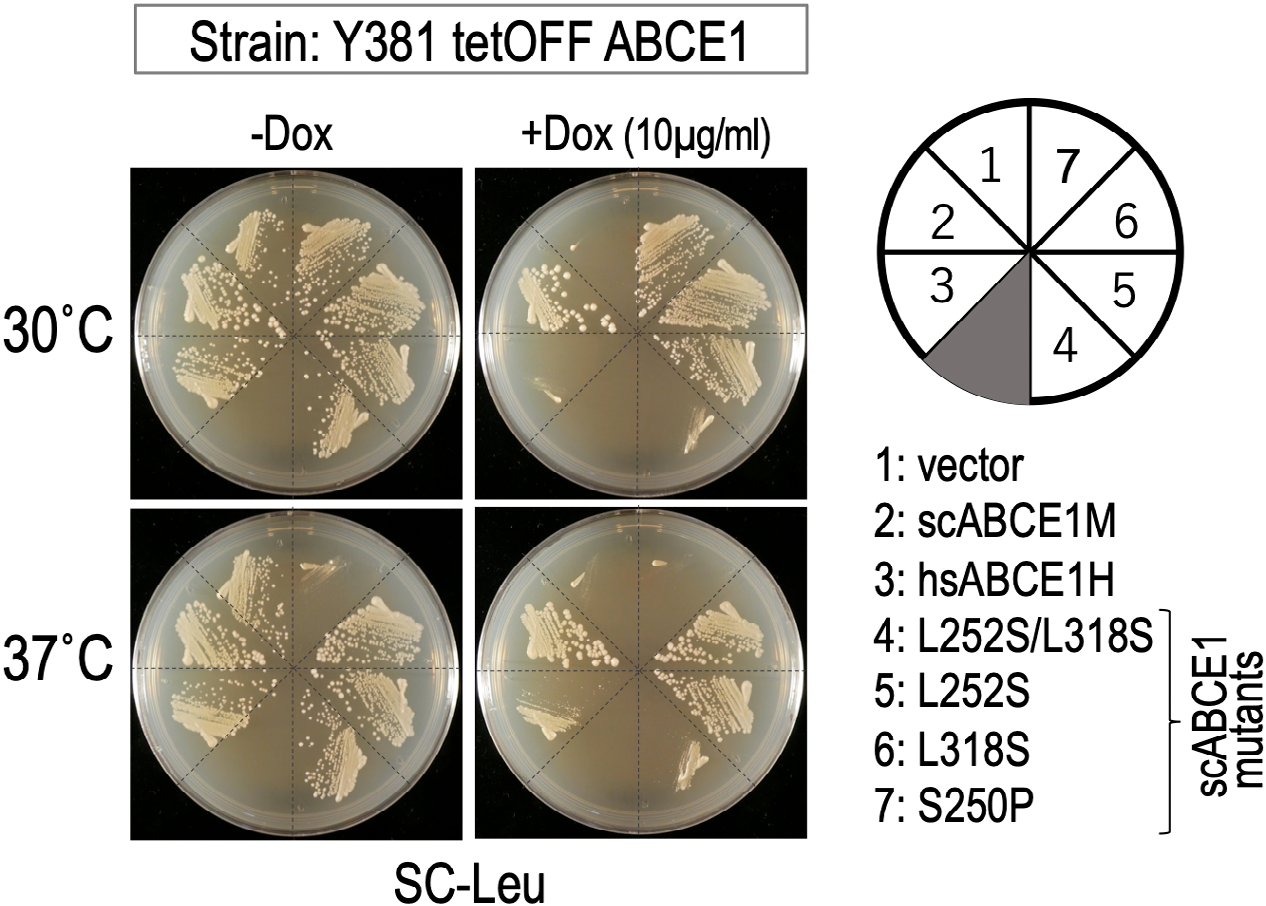
Differential effect of scABCE1 mutants and hsABCE1 in *S. cerevisiae*. The plasmids, p415ADH, scABCE1M and hsABCE1H, and scABCE1 mutants in p415GPD vector, L252S/L318S (double), L252S, L318S and S250P, were introduced into Y381 tetOFF ABCE1 strain. They were examined for growth on control SC‐L plates (left) and SC‐L plates containing 10 μg·mL^−1^ dox (right) as being indicative of functionality at 30 and 37 °C. The effect check experiments were performed in duplicate and repeated twice.

For reference to temperature‐dependent growth by hsABCE1 expression, isolation of temperature‐sensitive scABCE1 mutants was attempted in plasmid. Two interesting mutants were selected as related mutants, L252S/L318S and L250S, but not real temperature‐sensitive mutants. When L252S/L318S double mutation ABCE1 was expressed in Y381 tetOFF strain, the transformant showed slight growth at 30 °C and 37 °C in the presence of dox, the knockout condition, but each single mutant L252S and L318S transformant did not show growth defects (Fig. 4). The other mutant, L250S, showed a dominant negative phenotype at 37 °C. Y381 transformant of L250S ABCE1 showed growth at 30 °C in the presence or absence of WT ABCE1 expression. By contrast, at 37 °C, it did not grow in the presence or absence of WT ABCE1 expression, the knockout condition. The rather unusual behavior of L250S mutant is quite interesting, concomitantly with the temperature‐dependent functionality of hsABCE1 (Figs S1 and S2).

## Discussion

As a ribosome recycling factor, ABCE1 interacts closely with the ribosomes. It is reported to interact with translation termination factor eRF1 in a ribosome [6] and, as a result of large conformational change, it is retained in the 40S subunit of the ribosome [5, 9]. Considering the close interaction of ABCE1 with translation machinery, heterologous hsABCE1 should harmonize with the machinery of *S. cerevisiae* for function. On the other hand, as a human factor with two ATPase domains, hsABCE1 is expected to have higher activity at 37 °C. The higher activity of hsABCE1 may enable function in *S. cerevisiae* at 37 °C. In our previous study, co‐functional mutations were isolated from translation termination factor eRF3 of *Pneumocystis carinii* (a fungus), which is otherwise non‐functional in *S. cerevisiae* [10]. Many of the eRF3 mutations were considered to enhance mobility but not static interaction to gain functionality in *S. cerevisiae*. Taking this into account, heterologous hsABCE1 may provide higher functional flexibility at its original optimal temperature to overcome mismatch between heterologous co‐functional factors.

hsABCE1 and scABCE1 retain high homology around ATP/ADP binding sites and related areas, as well as FeS domain (Fig. S1). Many of the non‐conserved amino acids between them are located at the non‐ribosomal interaction side observed in 80S subunit interacting structures (Fig. S2). Detailed interaction partner(s) associated with co‐functionality are very interesting in relation to temperature‐dependency. In *S. cerevisiae*, temperature effects at the molecular level have been investigated as heat‐shock or temperature sensitive mutants. The finding of temperature‐dependent heterologous functionality of hsABCE1 may contribute to a better understanding of temperature effects at the molecular level.

## Conflicts of interest

The authors declare that they have no conflicts of interest.

## Author contributions

MW and KI conceived and supervised the study and performed the experiments.

## Supporting information


**Fig. S1.** Alignment of scABCE1 and hsABCE1 amino acid sequences. The sequences were aligned by uniprot (https://www.uniprot.org).
**Fig. S2.** Schematic drawing of ABCE1 structure (*Oryctolagus cuniculus*) with scABCE1 mutations and motifs indicated by homology. ABCE1 structure is derived from PDB: 3JAI. Non‐conserved amino acids between scABCE1 and hsABCE1 are shown in green. scABCE1 mutation sites are shown as red spheres. The ATP/ADP binding motifs and the signature motifs are shown in orange and yellow, respectively. The 3D structure was visualized using PyMol (The PyMOL Molecular Graphics System; Schrödinger, LLC, New York, NY, USA).

## Data Availability

The data are available from the corresponding authors on reasonable request.
