# Supplementary material for: Human ABCE1 exhibits temperature‐dependent heterologous co‐functionality in S. cerevisiae
Source: FEBS Open Bio. 2022 Jul 19;12(10):1782–7. doi: 10.1002/2211-5463.13463 (PMC9527587; doi:10.1002/2211-5463.13463)
Supplement: Supplementary file 1 — Fig. S1. Alignment of scABCE1 and hsABCE1 amino acid sequences. The sequences were aligned by uniprot (https://www.uniprot.org). Fig. S2. Schematic drawing of ABCE1 structure (Oryctolagus cuniculus) with scABCE1 mutations and motifs indicated by homology. ABCE1 structure is derived from PDB: 3JAI. Non‐conserved amino acids between scABCE1 and hsABCE1 are shown in green. scABCE1 mutation sites are shown as red spheres. The ATP/ADP binding motifs and the signature motifs are shown in orange and yellow, respectively. The 3D structure was visualized using PyMol (The PyMOL Molecular Graphics System; Schrödinger, LLC, New York, NY, USA). [file FEB4-12-1782-s001.pdf]

Supporting Information

Temperature-dependent Heterologous Co-functionality of Human ABCE1 in *S. cerevisiae*

Miki Wada* and Koichi Ito*

Fig. S1 Alignment of scABCE1 and hsABCE1 amino acid sequences.

Fig. S2 Schematic drawing of ABCE1 structure (*Oryctolagus cuniculus*).

```
HsABCE1-P.seq     1 MADKLTRIAIVNHDKCKPKKCRQECKKSCPVVRMGKLCIEVTPQSKIAWISETLCIGCGICIKKCPFGAL   70
ScABCE1-P.seq     1 MSDKNSRIAIVSADKCKPKKCRQECKRSCPVVKTGKLCIEVTPTSKIAFISEILCIGCGICVKKCPFDAI   70
                    * **  *****  ************* *****  ********* **** *** ******** ***** *
                                              FeS domain

HsABCE1-P.seq    71 SIVNLPSNLEKETTHRYCANAFKLHRLPIPRPGEVLGLVGTNGIGKSTALKILAGKQKPNLGKYDDPPDW  140
ScABCE1-P.seq    71 QIINLPTNLEAHVTHRYSANSFKLHRLPTPRPGQVLGLVGTNGIGKSTALKILAGKQKPNLGRFDDPPEW  140
                     * *** ***   **** ** ******* **** ****************************  **** *

HsABCE1-P.seq   141 QEILTYFRGSELQNYFTKILEDDLKAIIKPQYVDQIPKAAKG---TVGSILDRKDETKTQAI--VCQQLD  205
ScABCE1-P.seq   141 QEIIKYFRGSELQNYFTKMLEDDIKAIIKPQYVDNIPRAIKGPVQKVGELLKLRMEKSPEDVKRYIKILQ  210
                    ***  ************* **** ********** ** * **    **  *    *            *

HsABCE1-P.seq   206 LTHLKERNVEDLSGGELQRFACAVVCIQKADIFMFDEPSSYLDVKQRLKAAITIRSLINPDRYIIVVEHD  275
ScABCE1-P.seq   211 LENVLKRDIEKLSGGELQRFAIGMSCVQEADVYMFDEPSSYLDVKQRLNAAQIIRSLLAPTKYVICVEHD  280
                    *     *  * **********    * * **  *************** **  ****  *  * * ****

HsABCE1-P.seq   276 LSVLDYLSDFICCLYGVPSAYGVVTMPFSVREGINIFLDGYVPTENLRFRDASLVFKVAETANEEEVKKM  345
ScABCE1-P.seq   281 LSVLDYLSDFVCIIYGVPSVYGVVTLPASVREGINIFLDGHIPAENLRFRTEALQFRIADATEDLQNDSA  350
                    ********** *  ***** ***** * ************  * ******   * *  *

HsABCE1-P.seq   346 C-MYKYPGMKKKMGEFELAIVAGEFTDSEIMVMLGENGTGKTTFIRMLAGRLKPDEGGEVPVLNVSYKPQ  414
ScABCE1-P.seq   351 SRAFSYPSLKKTQGDFVLNVEEGEFSDSEILVMMGENGTGKTTLIKLLAGALKPDEGQDIPKLNVSMKPQ  420
                         **  **  * * *    *** **** ** ********* *  *** ******   * **** ***

HsABCE1-P.seq   415 KISPKSTGSVRQLLHEKIRDAYTHPQFVTDVMKPLQIENIIDQEVQTLSGGELQRVALALCLGKPADVYL  484
ScABCE1-P.seq   421 KIAPKFPGTVRQLFFKKIRGQFLNPQFQTDVVKPLRIDDIIDQEVQHLSGGELQRVAIVLALGIPADIYL  490
                    ** **  * ****   ***     *** *** *** *  ******* **********  * ** *** **

HsABCE1-P.seq   485 IDEPSAYLDSEQRLMAARVVKRFILHAKKTAFVVEHDFIMATYLADRVIVFDGVPSKNTVANSPQTLLAG  554
ScABCE1-P.seq   491 IDEPSAYLDSEQRIICSKVIRRFILHNKKTAFIVEHDFIMATYLADKVIVFEGIPSKNAHARAPESLLTG  560
                    *************     *  ***** ***** ************* **** * ****  *  *  ** *

HsABCE1-P.seq   555 MNKFLSQLEITFRRDPNNYRPRINKLNSIKDVEQKKSGNYFFLDD---                        599
ScABCE1-P.seq   561 CNRFLKNLNVTFRRDPNSFRPRINKLDSQMDKEQKSSGNYFFLDNTGI                        608
                     * **  *  *******  ******* *  * *** ********
```

scABCE1 mutation

FeS domain

ATP/ADP binding motif

Signature motif

Fig. S1 Alignment of scABCE1 and hsABCE1 amino acid sequences.
The sequences were aligned by uniprot (https://www.uniprot.org).

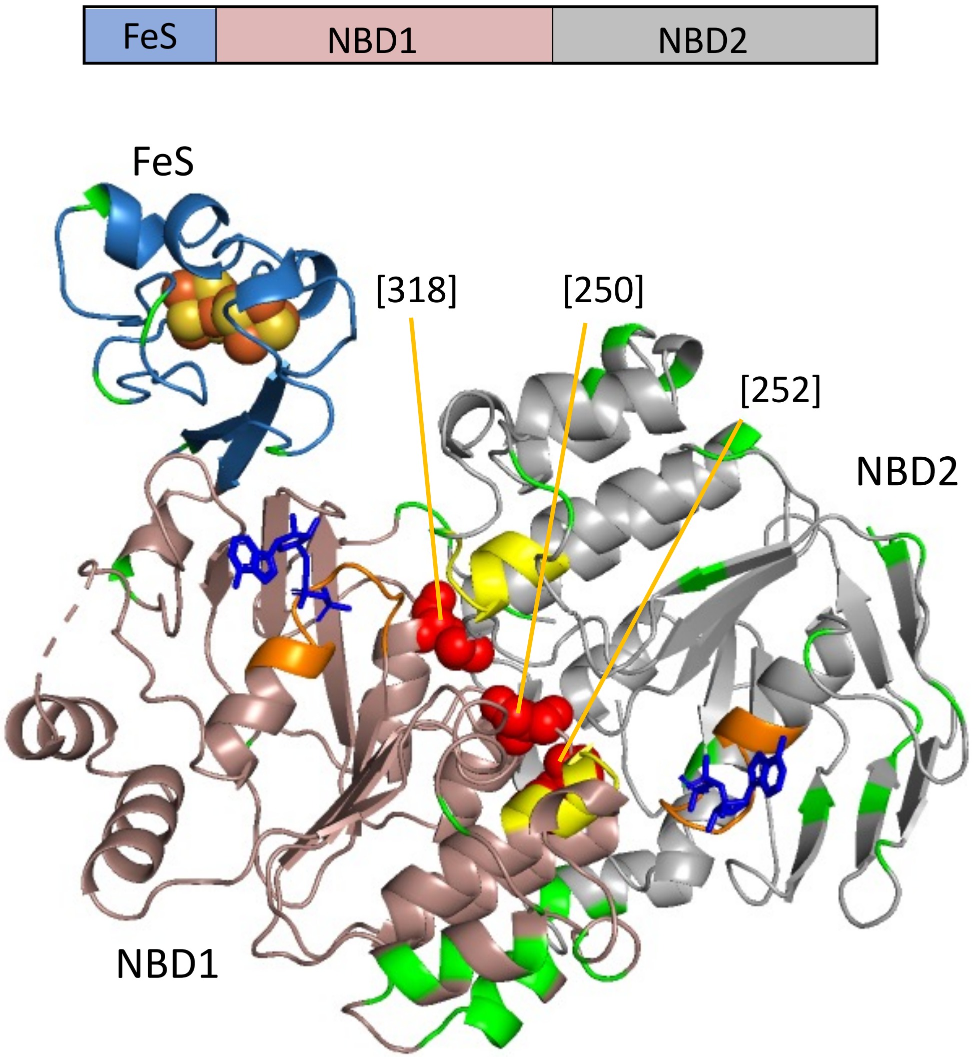

Fig. S2 Schematic drawing of ABCE1 structure (*Oryctolagus cuniculus*). The scABCE1 mutations and motifs are indicated by homology. ABCE1 structure is derived from PDB:3jai. Non-conserved amino acids between scABCE1 and hsABCE1 are shown in green. scABCE1 mutation sites are shown as red spheres. The ATP/ADP binding motifs and the signature motifs are shown in orange and yellow, respectively. The 3D structure was visualized using the PyMol Molecular Graphics System.
